# Vapor Phase Synthesis of SnS Facilitated by Ligand-Driven “Launch Vehicle” Effect in Tin Precursors

**DOI:** 10.3390/molecules26175367

**Published:** 2021-09-03

**Authors:** Ufuk Atamtürk, Veronika Brune, Shashank Mishra, Sanjay Mathur

**Affiliations:** 1 Institute of Inorganic Chemistry, University of Cologne, Greinstraße 6, 50939 Cologne, Germany; ufuk.atamtuerk01@gmail.com (U.A.); vbrune@uni-koeln.de (V.B.); 2 Institut de Recherches sur la Catalyse et l’Environnement de Lyon (IRCELYON), Université Claude Bernard Lyon 1, CNRS, UMR 5256, 2 Avenue Albert Einstein, 69626 Villeurbanne, France

**Keywords:** single-source precursor, chemical vapor deposition, tin sulfide, tertiary butyl sulfide, single-crystal structure

## Abstract

Extraordinary low-temperature vapor-phase synthesis of SnS thin films from single molecular precursors is attractive over conventional high-temperature solid-state methods. Molecular-level processing of functional materials is accompanied by several intrinsic advantages such as precise control over stoichiometry, phase selective synthesis, and uniform substrate coverage. We report here on the synthesis of a new heteroleptic molecular precursor containing (i) a thiolate ligand forming a direct Sn-S bond, and (ii) a chelating O^N^N-donor ligand introducing a “launch vehicle”-effect into the synthesized compound, thus remarkably increasing its volatility. The newly synthesized tin compound [Sn(SBu*^t^*)(*tfb-dmeda*)] **1** was characterized by single-crystal X-ray diffraction analysis that verified the desired Sn:S ratio in the molecule, which was demonstrated in the direct conversion of the molecular complex into SnS thin films. The multi-nuclei (^1^H, ^13^C, ^19^F, and ^119^Sn) and variable-temperature 1D and 2D NMR studies indicate retention of the overall solid-state structure of **1** in the solution and suggest the presence of a dynamic conformational equilibrium. The fragmentation behavior of **1** was analyzed by mass spectrometry and compared with those of homoleptic tin tertiary butyl thiolates [Sn(SBu*^t^*)_2_] and [Sn(SBu*^t^*)_4_]. The precursor **1** was then used to deposit SnS thin films on different substrates (FTO, Mo-coated soda-lime glass) by CVD and film growth rates at different temperatures (300–450 °C) and times (15–60 min), film thickness, crystalline quality, and surface morphology were investigated.

## 1. Introduction

The monochalcogenide material SnS, which is isostructural to black phosphorous [1], has recently gained much attention due to its unique semiconductor and optoelectronic properties, as well as its low toxicity and the earth-abundant character of tin and sulfur [2,3,4,5]. Two-dimensional (2D) layered monochalcogenides like SnS exhibit a puckered lattice structure with reduced crystal symmetry (orthorhombic) [6] and display structural resemblance to the classical 2D van der Waals material graphene and most of the transition metal dichalcogenides (TMDCs) [7,8]. Their layer-dependent tunable bandgap, high carrier mobility, and strong in-plane anisotropy make these materials promising for emerging next-generation electronic [9] and photonic applications [10,11]. SnS has been actively studied due to its p-type semiconductor characteristics and remarkable optoelectronic properties [12] due to the reported direct bandgap of about 1.3 and indirect bandgap of about 1.0 eV [13].

Various crystal structures are known for SnS, such as orthorhombic [3], zinc blende like [14], or a highly distorted rock salt (NaCl) structure [15], among which the orthorhombic is the most commonly found structure in SnS films reported so far [16]. Thin films of SnS have been obtained using various techniques such as thermal evaporation, chemical vapor deposition, electro-deposition, spray pyrolysis [17], and sulfurization of metallic precursors [18]. To achieve a stoichiometrically pure phase with desired material characteristics (morphology, grain size, etc.), carefully designed and optimized synthesis of the materials is necessary. Phase deposition processes like chemical vapor deposition (CVD) enable the growth of high-quality 2D materials with mono- or few-layer thickness control [19]. The preparation of SnS by CVD using multi-source precursors (MSP) has been reported previously, for instance, by reacting SnCl_4_ with H_2_S [20] or (PhS)_4_Sn with H_2_S [21]. However, the MSP approach requires at least two components possessing different properties like vapor pressure and stability in the gas phase and often uses hazardous co-reagent like H_2_S. Some of these drawbacks can be overcome by the single-source precursor (SSP) approach, which enables a predefined stoichiometry and phase control already at the molecular level. For instance, the tin(II) aminothiolate single-source precursor [Sn(SCH(Me)_2_CH_2_CH_2_NMe_2_)] was used for the deposition of SnS films with preferred crystal orientation along the (040) planes at temperatures as low as 300–350 °C [22]. The tin(II) dithiolate [Sn(SCH_2_CH_2_CH_2_S)_2_] has been used for phase-pure SnS deposition, obtained in MOCVD at temperatures between 300 and 400 °C [23]. The alkyltin chalcogenolate precursors [SnBu*^n^*_2_(SBu*^n^*)_2_] and [SnBu*^n^*_3_(SBu*^n^*)] were employed in low pressure (LP)-MOCVD for the deposition of phase pure SnS in the temperature range between 375 and 530 °C [24]. Aerosol-assisted chemical vapor deposition (AACVD) of tin(II) thioamidates [25], tin(II) thioureide, tin(II) dithiocarbamates [Sn(S_2_CNRR^’^)_2_] (R,R^’^ = alkyl) [26], tin(IV) dialkyldithiocarbamates [Sn(C_4_H_9_)(S_2_CNRR^’^)_2_], [Sn(C_6_H_9_)(S_2_CNRR^’^)_2_] [27] and heteroleptic tribenzyltin(IV) thiosemicarbazones Bz_3_SnCl(L) (L = thiosemicarbazone) [28] resulted in temperature-dependent polymorphs of SnS [29,30]. Using atmospheric pressure CVD (APCVD) and H_2_S with the asymmetric tin-dithiocarbamates [Sn(R)_2_(S_2_CNR^’^R^”^)_2_], a mixture of SnS and Sn_2_S_3_ was obtained [31]. Similarly, [Sn(SCH_2_CF_3_)_4_] and [Sn(SCH_2_CH_2_S)_2_] were employed for the deposition of SnS at 550 and 600 °C, respectively [32,33].

In many of the above-mentioned compounds, the presence of various element combinations introduces higher ionicity in the bonds, which often leads to higher sublimation temperatures that affect the thermal decomposition profile, leading to multi-step decomposition processes and concomitant formation of secondary phases. Further approaches to circumvent limited volatility include the application of a carrier gas, which is challenging for process engineering and reproducibility. Difficulties may also arise from a mismatch in oxidation states between the metal center (Sn(II) vs. Sn(IV) in the precursor and the final material. Herein, we present the direct synthesis of phase-pure SnS by CVD experiments performed at extraordinary low precursor temperatures (T_s_) using the SSP approach. By introducing a tridentate O^N^N ligand *Htfb-dmeda* [(3*Z*)-4-{[2-(dimethylamino)ethyl]amino}-1,1,1-trifluorobut-3-en-2-one] in pre-synthesized [Sn(SBu*^t^*)_2_], a monomeric and highly volatile tin(II) complex [Sn(SBu*^t^*)(*tfb-dmeda*)] **1** was obtained, which shows a clean and well-defined decomposition profile and sublimes without leaving any residue. The oxidation state of the Sn^II^ metal center remains unaltered and the element ratio Sn:S 1:1 is the same in the precursor molecule and in the desired final material SnS. The neutral role of the tridentate ligand *tfb-dmeda^−^* in the steric stabilization of the complex and its non-participating nature in the thermal decomposition process (no incorporation of N or O in the final products) makes it an ideal “launch vehicle” to deliver Sn and S atoms on the substrate surface during the CVD process.

## 2. Results and Discussion

### 2.1. Synthesis and Characterization of New Molecular Precursor

**Scheme 1:**
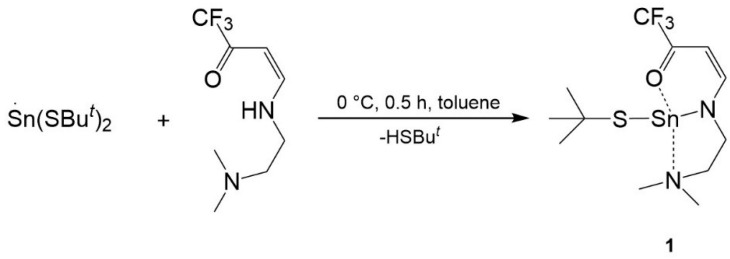
Synthesis of **1**.

The motivation of this work was to generate single-phase, nanostructured tin monosulfide SnS by CVD method using an extraordinary volatile molecular precursor. The monoanionic, tridentate coordinating *Htfb-dmeda* ligand was synthesized following the literature [34], whereas [Sn(SBu*^t^*)_2_] was synthesized using a simple thiolysis reaction between [Sn{N(SiMe_3_)}_2_] [35] and commercially available HSBu*^t^* (2-methyl-2-propanethiol). An equivalent amount of the ligand *Htfb-dmeda* was added to a toluene solution of [Sn(SBu*^t^*)_2_] under daylight exclusion, resulting in an orange solution. Removal of volatile compounds and purification of the orange product by sublimation enabled the isolation of the pure compound [Sn(SBu*^t^*)(*tfb-dmeda*)] **1** (Scheme 1).

#### 2.1.1. Single Crystal X-ray Diffraction Analysis of **1**

**Figure 1:**
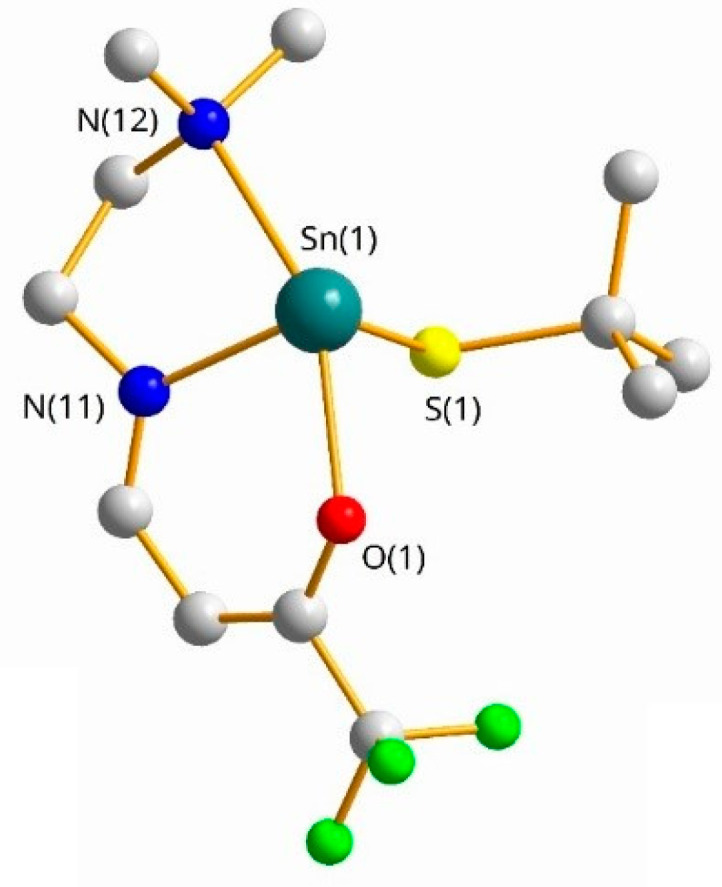
Molecular structure of [Sn(SBu*^t^*)(*tfb-dmeda*)] **1**.

Crystals of **1** suitable for single-crystal X-ray diffraction were obtained by sublimation. Compound **1** crystallized in the triclinic space group *P*$\bar{1}$ with four identical molecules [Sn(SBu*^t^*)(*tfb-dmeda*)] present in the asymmetric unit. Since there are no significant interactions present among these four independent molecules to form a pseudotetramer, only one such molecule is shown in Figure 1. The central Sn(II) atom in **1** is coordinated by one monodentate {SBu*^t^*}^−^ and one tridentate {tfb-dmeda}^−^ unit, resulting in a distorted (pseudo)trigonal-bipyramidal coordination geometry for it (Figure 1). Three equatorial positions are occupied by sulfur S(1), amine nitrogen N(11) and the lone pair of the Sn^II^ metal center. The axial positions are occupied by the carbonyl oxygen O(1) and the nitrogen atoms N(12) of the terminal -NMe_2_ group. Some selected bond lengths and angles are listed in Tables S1 and S2, respectively, in the Supporting Information.

In the basal plane, the N(11)-Sn(1)-S(1) bond angles [83.12(6)-84.93(7)°] are more compressed, as compared to the bond angles between the equatorial and apical positions, i.e., N(12)-Sn(1)-S(1) [86.47(7)-88.26(7)°] and O(1)-Sn(1)-S(1) [90.77(6)-92.53(6)°]. The {*tfb-dmeda*}^−^ ligand framework enforces a bent arrangement of the moiety with the bipyramidal axis O(1)-Sn(1)-N(12) [153.01(8)-154.04(8)°] deviating significantly from the ideal linear geometry. All the Sn-S, Sn-N, and Sn-O distances in **1** are well within the sum of their Van-der-Waals radii [36], and on the other hand, exceed the sum of the covalent radii for the corresponding element combinations [*r*_cov_(Sn) = 140 pm, *r*_cov_(N) = 71 pm, *r*_cov_(O) = 63 pm, *r*_cov_(S) = 103 pm] [37].

The Sn-S distances in **1** [248.61(8)–249.27(8) pm] are only slightly above their corresponding covalent radii sum ∑_cov_(Sn-S = 243 pm) bearing a high tendency towards covalency in their bonds. These tin–sulfur Sn-S bond lengths in **1** are shorter than those reported for the homoleptic tin(II) alkylthiolates [38,39,40,41,42,43]. Compound [Sn(SBu*^t^*)_2_]*_n_* [38] possesses two shorter Sn-S bonds [Sn-S = 252.7(2) and 252.9(2) pm] and two longer Sn←S coordinative bonds [Sn-S = 303.4(2) and 303.3(2) pm], resulting in a network of interlinked molecular units with four-fold coordinated Sn^II^ atoms in *pseudo*-trigonal bipyramidal geometry. Similarly, in the 1D coordination polymer [Sn(SPh)_2_]*_n_*, the central tin(II) atom adopts a distorted trigonal pyramidal coordination geometry, with two shorter Sn-S bonds [251.8(2) and 257.7(2) pm] and two longer Sn←S coordinative bonds [273.1(2) pm] [42]. With bulkier substituents, lower degrees of oligomerization are realized as observed in the monomeric compounds [Sn(SMes*)_2_] [Sn-S = 243.56(3) pm] [43] and [Sn(S-2,4,6-*^t^*C_4_H_9_C_6_H_2_)_2_] [39] [Sn-S = 243.5(1) pm] or in the trimeric [Sn(S-2,6-*^i^*C_3_H_7_C_6_H_2_)_2_]_3_ [Sn-S = 247.1(5)–283.8(4) pm] [39].

In anionic molecular tin(II) compounds, the Sn-S bond lengths show minor differences as compared to those in the coordination polymers, such as [(Me_3_Si)_3_CSn(*µ*-SBu)_2_]^−^ [41] [Sn-S = 256.8(1)–257.4(1) pm] or [Sn(SPh)_3_]^−^ [44] [Sn-S = 253.2(1) and 255.2(1) pm]. In analogous tin(IV) compounds, the tendency to form coordination polymers is absent. For example, [Sn(SBu*^t^*)_4_] [Sn–S = 239.7(2) pm] [45] and [Sn(SPh)_4_] [Sn-S = 237.9(4) and 240.1(4) pm] [21] exist in their solid state structures as monomeric units with distorted tetrahedral geometry around the tin(IV) metal center.

The central tin(II) atom interacts with both the N(11) and N(12) nitrogen atoms in **1** by Sn←N coordinative bonds. One set of larger Sn(1)-N(12) distances in **1** [Sn-N = 254.1(3)–255.7(3) pm] arises from weak intramolecular Sn←N coordination of the terminal -NMe_2_ group. The second set of significantly shorter Sn-N distances Sn(1)-N(11) [Sn-N: 224.2(2)–226.0(2) pm] can be categorized as stronger Sn←N coordinative interaction, which are comparable to the bonding strength displayed by *µ*-bridging monoanionic nitrogen donor atoms, as found in the dimeric [Sn(NMe_2_)_2_]_2_ with two terminal Sn-N single bonds [Sn-N = 206.7(4) pm] and two bridging Sn-*µ*N [Sn-N = 226.5(4) and 226.6(5) pm] [46].

For comparison, the Sn(1)-N(12) distances in **1** are longer than in the monomeric [Sn(OCH_2_CH_2_NMe_2_)_2_] [Sn-N = 245.8(2) pm] [47], but shorter than the value observed in the heteroleptic dimer [{(Me_3_Si)_2_N}Sn(OCH_2_CH_2_NMe_2_)]_2_ [40] with weaker Sn←N coordinative interaction between the tin(II) center and the -NMe_2_ moieties [Sn-N = 261.5(3) and 261.7(3) pm]. Similarly, a Sn^II^←O coordinative bonding interaction between the central tin(II) atom and the carbonyl oxygen atom is present in **1**, with Sn-O distances typically observed between tin(II) metal centers and neutral oxygen donor atoms [48,49]. The experimental distances Sn-O in **1** [Sn-O = 223.7(2)–226.9(2) pm] fall in the region of bond lengths measured for *µ*-bridging anionic oxygen donor atoms or intermolecular contacts. Corresponding Sn-O single bond lengths between tin(II) and an anionic oxygen donor atom are significantly shorter. For example, the Sn-O bond lengths lie in the range 212.8(4)–216.5(4) pm in [Sn(OBu*^t^*)_2_]_2_, having an archetypical “*Sn_2_O_2_”* core as a structural motif [38]. One Sn-O single bond length is reported for [Sn(OCH_2_CH_2_NMe_2_)_2_] [Sn-O = 205.6(2) pm] [47], and in [{(Me_3_Si)_2_N}Sn(OCH_2_CH_2_NMe_2_)]_2_, two Sn-O single bonds [Sn-O = 213.3(2) and 213.7(2) pm] and two Sn←O coordinative bonds [Sn-O = 226.7(2) and 229.9(2) pm] are observed [40].

In the heteroleptic tin(II) compound [Sn(SCH(Me)_2_CH_2_CH_2_NMe_2_)_2_] [22], the Sn-S bond [Sn-S = 252.63(4) pm] is elongated, whereas the Sn-N distance arising from the -NMe_2_ moiety [Sn-N = 247.13(12) pm] is shorter in comparison to **1**. Similar observations were made in [Sn(SC(Pr*^i^*)N(Bu*^t^*))_2_], where two bidentate thioimidates, each coordinated through sulfur and nitrogen atoms, form a distorted square-pyramid with the central tin(II) atom [Sn-N = 237.91(12) and Sn-S = 254.67(4) pm] [25]. The comparative observations above reveal the shortest nature of the Sn-S bond length in **1** among all the tin(II) alkylthiolates Sn(SR)_2_ reported so far.

#### 2.1.2. Spectroscopic Characterization of **1**

**Figure 2:**
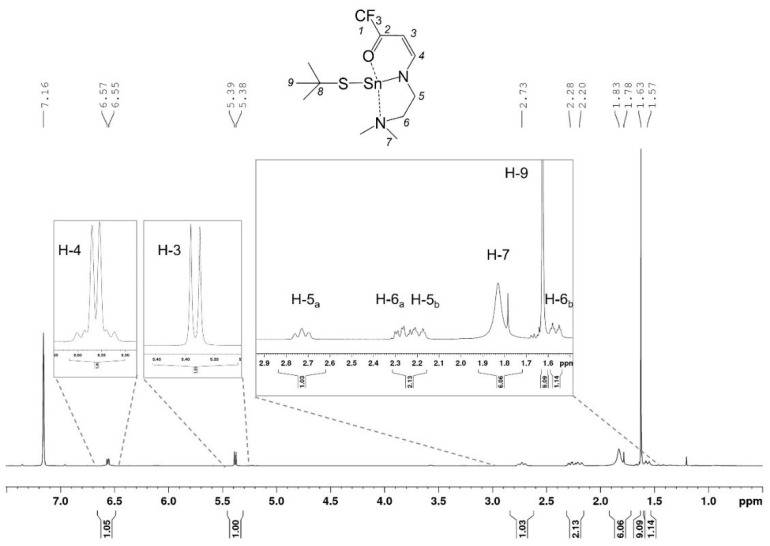
^1^H-NMR spectrum of **1** in C_6_D_6_ recorded at 400 MHz.

The structure of **1** in solution was analyzed by a combination of 1D and 2D NMR experiments of ^1^H, ^13^C, ^19^F, and ^119^Sn nuclei. The ^1^H-NMR spectrum of **1** in C_6_D_6_ solvent recorded at 400 MHz showed (Figure 2) the formation of a single reaction product that was also supported by the high yield of **1** obtained after sublimation, which excluded the simultaneous presence of other possible species such as fully substituted Sn atoms or oxidative addition product. As a result of deprotonation, the resonance of the (4-H) proton at *δ* = 6.56 becomes a doublet by vicinal coupling to (H-3). The associated dd (doublet of doublets) satellite signal showed the coupling between these protons and the ^119^Sn-nuclei (^3^J_1H-119Sn_ = 25 Hz).

The signal splitting of the methylene proton resonances in **1** from the saturated region (C-5) and (C-6) of the ligand molecule leads to four distinct chemical shifts *δ*^1^H. This is interpreted as a result of these protons being part of a more or less rigid N-C_5_-C_6_-N(CH_3_)_2_ framework, which formed a 5-membered ring by the coordination of the nitrogen atoms to the Sn metal center (Figure 1). Although clearly under the influence of second-order effects, and probably also by dynamic broadening, the not so fully-resolved multiplet of the (H-5_a_) and (H-5_b_) methylene protons (centered at *δ* = 2.73 and *δ* = 2.30, respectively) indicated a typical spin-system, that would arise from the relation between axial (endo) and equatorial (exo) protons of a ring structure. The resonance(s) of the methyl protons from the -N(CH_3_)_2_ group at *δ* = 1.83 displayed an increased linewidth (Δ_1/2_ = 25 Hz), as compared to the free protonated ligand (Figure S1), pointing towards solution dynamics occurring at rates far below than that of free rotation. The sharp signal at *δ* = 1.78 is attributed to the dynamic nature of -N(CH_3_)_2_ group, since both signal integrals add up to six protons. However, in the case of broad signals, this is not a very reliable argument, and its origin may not be conclusively explained within this study.

Upon cooling the solution of **1** in toluene-d_8_, the resonance at *δ* = 1.83 (H-7) underwent further broadening and flattened out at T = 268 K; thus the coalescence temperature was noted as T_c_ = 278 K. Below this temperature, separate signals for the exchanging conformers are expected to emerge (further cooling was intended but due to instruments conditions of the VT-unit the full capabilities of the spectrometers could not be fully exploited). In the observed temperature range, no significant shift or change in linewidth was observed (Figures S2 and S3).

In summary, the proton resonances from the unsaturated carbon moiety are not affected by a dynamic process, and coupling is observed between (H-4) and the central ^119^Sn nuclei. The methylene protons at the saturated (C-5) and (C-6) carbon atoms are chemically and magnetically not equivalent, and are part of a conformational equilibrium and by variable temperature (VT) NMR, we could determine the coalescence temperature T_c_ = 278 K of the -N(CH_3_)_2_ group, that shows fluxional behavior in solution.

The ^119^Sn{^1^H}-NMR spectrum at room temperature displayed a single broad resonance at *δ =* −157 (Figure S4). The value lies well within the chemical shift region (roughly from +150 to −400 ppm) identified with dimers of tin(II) present in a threefold coordination by sulfur, oxygen, and nitrogen donor atoms in varying element combinations [50]. Since these compounds become four coordinated in the dimer, there is a high resemblance to the bonding situation in **1**. In the above-mentioned chemical shift range, an identical set of donor atoms with identical coordinating geometry around the Sn^II^ metal center may be found separated by more than a hundred ppm, which shows the nature of the ligand to be also highly influential [50]. The chemical shift of [Sn(SBu*^t^*)_2_] is reported at *δ* = 77 (CH_2_Cl_2_) and 63 (THF) [51], which could not be observed, probably due to the fast solution dynamics observed in the reported signal’s linewidth of Δ_1/2_ = 1 kHz.

**Figure 3:**
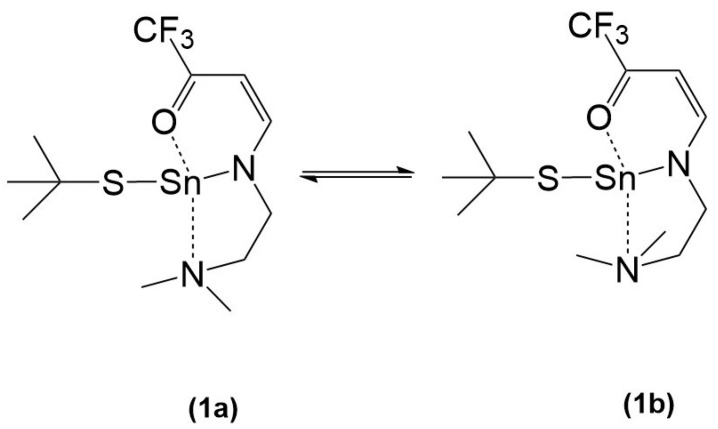
Proposed solution dynamics between two conformational isomers.

The broad linewidth of the *δ* ^119^Sn resonance (Δ_1/2_ = 300 Hz) in compound **1** indicated a dynamic exchange present at room temperature (Figure 3), supporting the observations made by the ^1^H-NMR investigations. Upon cooling the solution down to T = 268 K, the chemical shift was observed at *δ* = −167 (Δ_1/2_ = 200 Hz) with narrower linewidth (as compared to the one obtained at room temperature). This slight dependence of the chemical shift on temperature suggested a conformational equilibrium that does not involve changes in the coordination number or geometry of the ^119^Sn-nuclei [52]. The observation that in the presence of a nucleophilic solvent such as THF, the chemical shift *δ* ^119^Sn remained constant, showed a stable and saturated coordination sphere at the central Sn^II^ atom. Additionally, in the absence of a concentration-dependence of the *δ* ^119^Sn chemical shift, self-association processes such as aggregation or dimerization become unlikely [52,53,54]. Finally, the coupling between the central ^119^Sn nuclei and (H-6_a,b_) and (H-5_a,b_) methylene protons is shown by a ^119^Sn,^1^H-HMBC experiment (Figure S5).

To summarize the results from the NMR experiments, we showed that the structure of **1** observed in the solid state is maintained in solution and suggest the presence of dynamic conformational equilibrium in the temperature range between T = 298–268 K as displayed in Figure 3. If **1** is kept in solution for a prolonged duration, gradual formation of the oxidized product [Sn(SBu*^t^*)_4_] along with disulfide is observed. However, if stored under inert conditions, **1** remained stable without any detectable signs of degradation for several months.

**Figure 4:**
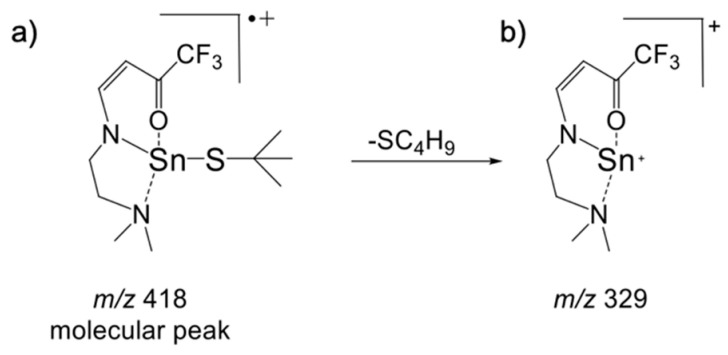
Scheme of the gas phase fragmentation of [*L*-Sn-SC_4_H_9_]^+^ (*m/z* = 418) (**a**) into [*L*-Sn]^+^ (*m/z* 329) species (**b**) under EI-MS conditions.

The fragmentation behavior of **1** was analyzed by mass spectrometry. For simplicity, the O^N^N-donor ligand moiety in compound **1** is abbreviated as *L* (*L= tfb-dmeda^−^*) and, therefore, **1** and its fragment notation as molecular peak are written as (*L*)-Sn-(SBu*^t^*) and [*L*-Sn-SC_4_H_9_]^+^ = [M]^+^, respectively. Mass spectrometric analysis showed that the molecule exists as a monomer in the gas phase, as identified by its molecular peak [M^+^] (*m/z* = 418) (Figure 4a). The signal of highest relative intensity belongs to the cationic “tin-ligand” species [*L*-Sn]^+^ (*m/z* 329, [M−SC_4_H_9_]^+^ (Figure 4b) as a result of the loss of the alkylthiolate radical fragment. The only signals that account for further fragmentation of [L-Sn]^+^ are found with the smallest ion fragments (*m/z* = 28 and 58, which are typical organic carbon-hetero atom(s) fragments in the different element combinations of C,H,N,O). In other words, the ligand L in [*L*-Sn]^+^ leaves the Sn-metal center in one piece prior to its own fragmentation. There is no fragment ion with a tin atom bound to oxygen or nitrogen, unlike the complex [Re(*tfb-dmeda*)(CO)_3_], where partial decomposition of the *tfb-dmeda* ligand while being bonded to the metal center has been observed by mass spectrometry [34].

**Scheme 2:**
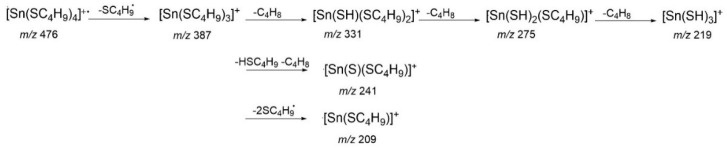
Fragment ions detected together with **1** by EI-MS, which are attributed to species occurring also in the EI-MS of [Sn(SBu*^t^*)_4_].

An initial fragmentation step of [M]^+^ that cleaves the bond between the ligand *L* and a complete *Sn-SC_4_H_9_* unit generates either one of the two fragments [*L*]^+^ (*m*/*z* 209) and [Sn-SC_4_H_9_]^+^ (*m/z* 209) that are, by coincidence, identical in their *m/z* values. The signal detected at *m/z* 209 is most likely [Sn-SC_4_H_9_]^+^, as explained further in the following text. The remaining signals could conclusively be assigned to tin alkylthiolate species (Scheme 2) which showed a high resemblance to the fragment ions previously reported for [Sn(SBu*^t^*)_4_] [55] and to [Sn(SBu*^t^*)_2_]. For the latter compound, no literature data was available, therefore, it was analyzed for comparison purposes in this work. All three compounds share at least partially a common decomposition pathway.

For [Sn(SBu*^t^*)_4_], the consecutive loss of alkyl groups in the decomposition pattern has been reported earlier [55]. The loss of alkyl groups in this context is equivalent to the preferred retainment of the Sn-S bond. From Scheme 2, this becomes obvious by the sheer number of fragments with a Sn-S bond present in the gas phase. Our mass spectrometric analysis showed that the fragmentation pattern of [Sn(SBu*^t^*)_4_] could partially be found also in the fragmentation pattern of [Sn(SBu*^t^*)_2_] (Figure S7). [Sn(SBu*^t^*)_2_] is, as [Sn(SR)_2_] (R = alkyl) in general, prone to oxidation upon heating. The experimental conditions, namely the sublimation step prior to fragmentation in the mass spectrometer, may explain the observation of tin(IV) species in the gas phase.

The observation was different for **1**, which is sublimable without decomposition or any other undesired side-reaction. This was confirmed by repeated resublimation, where **1** was retrieved quantitatively after each sublimation step, and its identity was confirmed by NMR spectroscopy of the sublimate. To exclude the presence of possible contamination by oxidized species, such as Sn(SBu*^t^*)_4_, the purity of **1** and Sn(SBu*^t^*)_2_ was confirmed by ^1^H- and ^119^Sn-NMR spectroscopy prior to the measurements.

An investigation on the exact mechanisms that proceed under EI conditions in the gas phase is beyond the scope of this work and was not further looked into. [Sn(SPh)_4_] and [Sn(SBu^t^)_4_] have been reported to yield Sn_2_O_3_ films by CVD [21,45]. Given the similarities observed here in the fragments of **1**, [Sn(SBu*^t^*)_4_] and [Sn(SBu*^t^*)_2_], the reported tin oxide formation by gas-phase deposition may likely not be an inherent property of the molecules, but rather due to experimental conditions such as the usage and nature of solvent during the aerosol-assisted CVD process. However, the application of [Sn(SR)_2_] and [Sn(SR)_4_] (R = alkyl, phenyl) for CVD remains limited since these compounds lack the desired sublimation properties.

#### 2.1.3. Thermogravimetric Analysis of **1**

**Figure 5:**
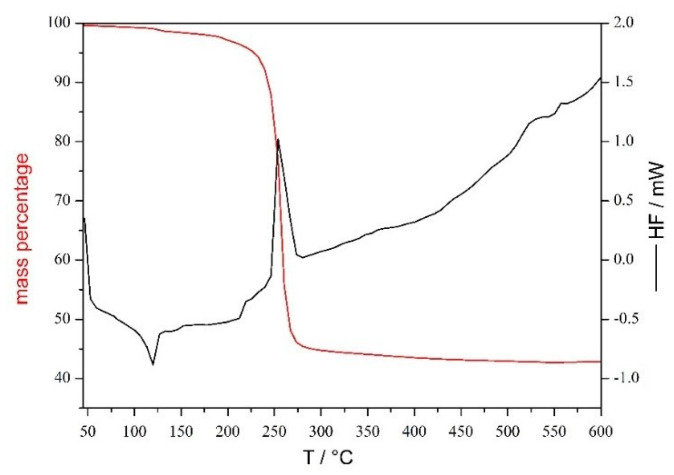
TG-DSC plot of the precursor [Sn(L-NMe_2_)(SBu^t^)] (**1**). The TGA curve is shown as red line, whereas the black line represents the DSC curve.

Thermogravimetric analysis was performed under continuous N_2_(g) flow to further investigate the decomposition behavior of **1** in relation to the temperature (Figure 5). Interestingly, the thermal decomposition of **1** occurred in one well-defined single step, as indicated by a steep mass loss in the TGA curve in the narrow temperature range between T = 250–275 °C. No significant mass difference or thermodynamic event was registered beyond 300°C that indicated the formation of a solid of definite composition. The experimental mass loss determined by TG-DSC is ∆m_exp._ = 57%, which is below the theoretical value ∆m_th._ = 64%, assuming the formation of SnS as the final product. One-step decomposition of **1** at remarkably low temperatures underlines its potential as a promising molecular precursor in CVD experiments.

### 2.2. CVD Experiments

The CVD experiments were conducted in a quartz glass tube fitted with an inductively heated graphite holder, where the substrates were mounted on. The precursor temperature (T_p_) for **1** was maintained between T_p_ = 90–130 °C and substrate temperatures chosen in this study were T_s_ = 300–450 °C that resulted in crystalline deposits of homogeneous SnS films.

**Figure 6:**
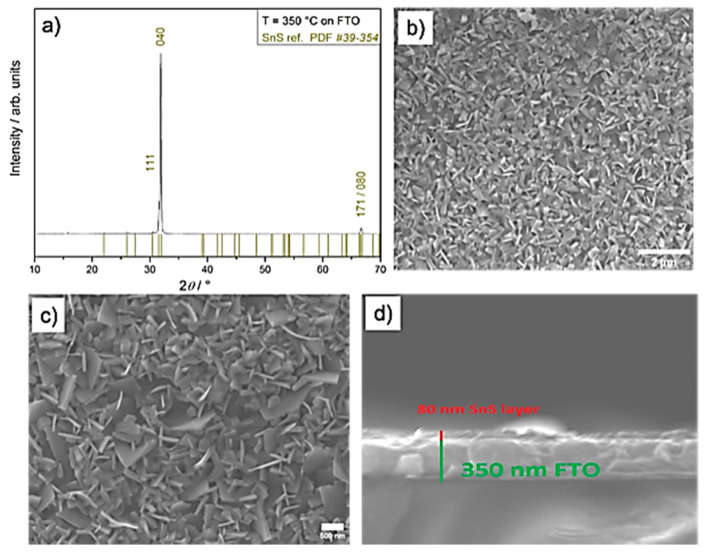
(**a**) GI-XRD (recorded in grazing incidence mode) pattern of SnS deposited on FTO substrate at T = 350 °C. (**b**,**c**) SEM top view of SnS on FTO, (**d**) SEM cross-section of SnS on FTO (SnS layer around 80 nm).

At precursor temperature of T_p_ = 130 °C and substrates maintained at 350 °C, the FTO substrate was uniformly covered by a black layer within 30 min of the processing time. The deposit was identified by XRD analysis to be SnS (Figure 6a) that exhibited diffraction peaks corresponding to (040) plane, along with a minor peak due to the (111)-plane. This showed the preferred orientation of grains in SnS films, namely along (040)-planes, parallel to the substrate surface. The SEM images (Figure 6b,c) showed the uniform coverage of the substrate surface by flake-like nanostructures. From the cross-sectional SEM (Figure 6d), the film thickness was measured to be ~80 nm. The (040) surface in SnS is the lowest energy surface [56] and, therefore, favored by kinetically limited growth conditions, such as lower temperatures and sufficiently long diffusion lengths. These low energy surfaces are bonded by van der Waals forces, and an orientation parallel to the substrate surface is favored by corresponding substrates, as previously reported [57]. On transition metals like molybdenum, covalent edge bonding is favored, resulting in (040) planes perpendicular and (101) surface parallel to the substrate surface, which results in the formation of plates growing out of the substrate plane [58]. The strong adhesion of the deposited SnS on FTO is possibly due to a different bonding situation than the aforementioned Van der Waals interaction, for instance, the formation of a mixed tin oxide-chalcogenide SnO_1−x_S_x_ phase at the interface between the substrate and the film. The mechanism that leads to the orientational growth on FTO is not yet fully understood and is subject to further investigations.

**Figure 7:**
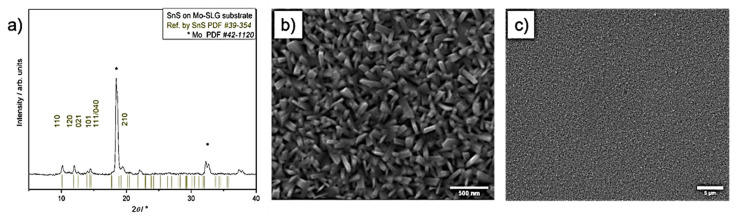
XRD pattern (**a**) of SnS deposited at 350 °C, and SEM top view at different magnifications (**b**,**c**) of SnS deposited on Mo-SLG at T = 350 °C.

In view of the application of SnS in photovoltaic devices and suitable band alignment, we chose to investigate the CVD process on molybdenum substrates. Herein, we use molybdenum layers of a uniform thickness that were sputtered on soda-lime glass (Mo-SLG). Under similar deposition conditions as described for the SnS deposition onto FTO (i.e., T_p_ = 130 °C, T_s_ = 350 °C), successful deposition of the crystalline SnS was observed onto Mo-SLG substrates, as confirmed by XRD measurements (Figure 7a). The SEM investigation of as-deposited films showed full coverage of the substrate (Figure 7 b,c).

**Figure 8:**
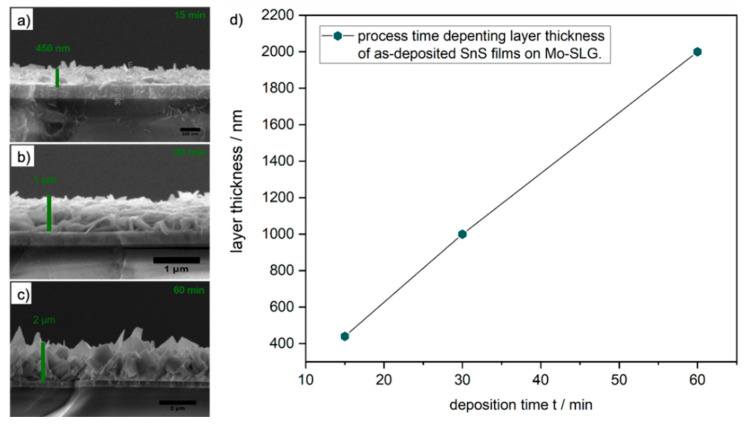
Cross-sectional view on the SnS layer thickness obtained after (**a**) 15 min, (**b**) 30 min, (**c**) 60 min, and (**d**) plot of time-depending layer thickness of as-deposited SnS films.

**Figure 9:**
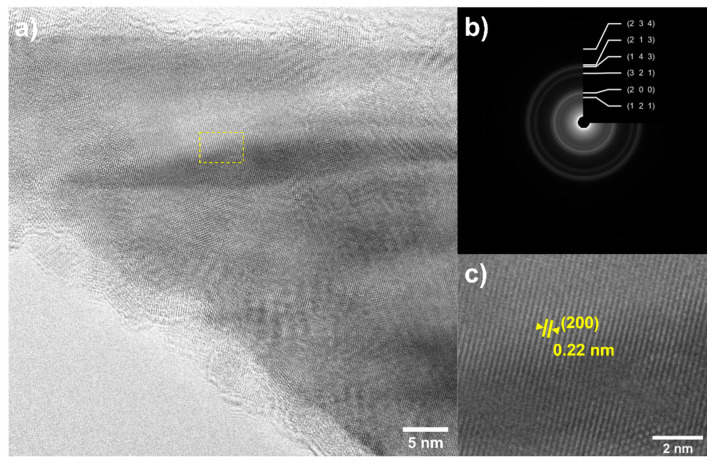
(**a**) TEM image of SnS scratched off from the films deposited on molybdenium substrate at T = 350 °C for 15 min, (**b**) ring diffraction pattern (upper inset), and (**c**) HRTEM image showing the (200) planes of the orthorhombic SnS (lower inset).

The film thickness was controlled by the process time (Figure 8a–c). By varying deposition times (15, 30, and 60 min), a linear relationship between the growth rate and deposition time was established (Figure 8d). The thicknesses were measured as 440 nm, 1 µm and 2 µm, thus, in the given process conditions the layers grew around 33 nm per minute, which is in good agreement with the film growth reported in the literature before [57]. The different SnS films, thicknesses were measured using SEM cross-sectional analyses (Figure 8a–c). The low dimensional platelets of SnS were found to be vertically oriented to the substrate surface, as clearly seen in Figure 8c. This morphological attribute offers a huge number of active sites of the layered SnS phase that consequently increases the catalytic behavior of as-deposit material potentially interesting for the application in photovoltaic systems [59]. To analyze the films further by high-resolution transmission electron microscopy (HRTEM) and selected area electron diffraction (SAED), parts of the film deposited on molybdenum substrate for 15 min at T = 350 °C were scratched off the substrate surface. The HRTEM image shows clear lattice fringes, therefore, further confirms the high crystallinity of the material (Figure 9). The diffraction spots in the SAED pattern are indexed consistently with the d-spacings of orthorhombic SnS (COD # 9008785). The indexed planes correspond to the [0 −1 2] zone axis presented as a ring diffraction pattern.

## 3. Materials and Methods

All experiments were carried out under strict exclusion of moisture and oxygen under an atmosphere of dry N_2_ (*g*) using stock high vacuum apparatus. Solvents were dried by refluxing over sodium and benzophenone and freshly distilled prior to use and stored over sodium wire. HSBu*^t^* (2-methyl-2-propanethiol) was purchased from Sigma-Aldrich (99%) and used without further purification. [Sn{N(SiMe_3_)_2_}_2_] was synthesized as reported earlier [35]. [Sn(SBu*^t^*)_2_] was synthesized via the thiolysis of the stannylene [Sn{N(SiMe_3_)}_2_] in toluene at T = 0 °C under exclusion of ambient light. After removing all volatile components, bright yellow needle-like crystals were obtained and used without further purification. The ligand *Htfb-dmeda* (3Z)-4-{[2-(dimethylamino)ethyl]amino}-1,1,1-trifluorobut-3-en-2-one, was provided by the working group members and checked by ^19^F-, ^13^C- and ^1^H-NMR prior to use. A detailed synthetic procedure for ligand is described in the reference [34].

NMR spectra were recorded on a Bruker (Billerica, MA, USA) Model Avance II 300 and Avance 400 spectrometer. ^1^H-NMR and ^13^C-NMR chemical shifts are reported in parts per million relative to external tetramethylsilane (TMS) and are referenced internally to residual proton impurity or ^13^C signal of the deuterated lock solvent. Assignment of all ^13^C-NMR signals was made on the basis of ^1^H,^13^C-HMQC, ^1^H,^13^C-HMBC, and ^19^F,^13^C-HMBC experiments. The ^119^Sn{^1^H} spectra were calibrated externally to [Sn{N(SiMe_3_)_2_}]_2_ [35] in C_6_D_6_ (δ^119^Sn = 775) and are reported relative to Sn(CH_3_)_4_ in C_6_D_6_ (*δ* = 0 ).

Elemental analyses were carried out on a HEKAtech CHNS Euro EA 3000 (Wegberg, Germany). TG-DSC measurements were performed on a TGA/DSC1 (Mettler-Toledo GmbH, Gießen, Germany) apparatus. Mass spectrometric data were recorded on a Finnigan MAT 95 (EI, 20 eV) and are reported as *m*/*z* (rel. intensity in %). High-resolution TEM (HRTEM) and SAED measurements were carried out on a JEOL JEM-2200FS microscope (Peabody, London, UK).

Data collection for X-ray structure elucidation was performed on a STOE IPDS 2T diffractometer (Darmstadt, Germany) using graphite-monochromated Mo-K_α_ radiation (0.71071 Å). The data were corrected for Lorentz and polarization effects. A numerical absorption correction based on crystal-shape optimization was applied for all data. The software used in this work are STOE’s X-Area, SHELXL [60,61], and ShelXle [62] (Version 93, Darmstadt, Germany). CCDC *2102248* contains the supplementary crystallographic data for this article. These data can be obtained free of charge from The Cambridge Crystallographic Data Centre, via www.ccdc.cam.ac.uk/data_request/cif, accessed on 10 August 2021.

The MOCVD experiments were conducted in a cold-wall CVD reactor under low-pressure conditions (*p* < 10^−6^ bar), equipped with cooling traps, an internal pressure sensor and external temperature sensors. More detailed information can be found in previous publications from our working group [63,64,65].

### Synthesis of [Sn^II^(tfb-dmeda)(SBu^t^)] (***1***)

To a solution of Htfb-dmeda (1.185 g, 5.640 mmol) in toluene (5 mL) was slowly added a suspension of bis(*tert*-butylthiolato)tin(II) (1.671 g, 5.624 mmol) in toluene (8 mL) at 0° by cooling with crushed ice. The reaction mixture soon changed into an orange solution. The cooling bath was removed, and the orange solution was stirred for 0.5 h under exclusion of ambient daylight. Removal of all volatile compounds *in vacuo* left an orange solid, which was further purified by sublimation (90–100 °C, 10^−3^ mbar). After sublimation, **1** was obtained as orange to yellow crystalline solid in 82% yield. Elemental analysis. Calcd. for C_12_H_21_F_3_N_2_OSSn: C, 34.55%; H, 5.09%; N, 6.72%; S, 7.69%. Found: C, 34.63% H, 5.09%; N, 6.97%; S, 8.23%.

**^1^H-NMR** (400.1 MHz, C_6_D_6_, 25 °C) δ = 6.58 (d, *J*_H-H_ = 7 Hz, ^3^*J*_1H-119Sn_ = 25 Hz, 1H, H-4) 5.39 (d, J_H-H_ = 7 Hz, 1H,H-3), 2.79–2.67 (m, 1H,H-5_a_), 2.33–2.23 (m, 1H,H-6_a_), 2.23–2.15 (m, 1H,H-5_b_), 1.83 (br, 6H,H-7), 1.78 (s, H-7), 1.63 (s, 9H, H-9), 1.60–1.52 (m, 1H,H-6_b_).

**^119^Sn{^1^H}-NMR** (149.2 MHz, C_6_D_6_, 25°C) δ = −159 (s, Δ_1/2_ = 300 Hz)

**VT-NMR:**

**^119^Sn{^1^H}-NMR** (149.2 MHz, C_7_D_8_) T= 298 K: −159 (s, Δ_1/2_ = 300 Hz). T= 288 K: −161 (s, Δ_1/2_ = 300 Hz), T= 278 K: −164 (s, Δ_1/2_ = 260 Hz), T= 268 K: −167 (s, Δ_1/2_ =200 Hz).

The proton resonances from the VT NMR experiment are not listed, since in the observed temperature range, the broadening of the (H-7) proton resonances from -N(Me)_2_ (*δ*^1^H = 1.83 at r.t.) was the only significant event, as explained in the text.

**^1^H-NMR** (300.1 MHz, C_6_D_6_, 25 °C) δ = 6.58 (d, *J*_H-H_ = 7 Hz, ^2^*J*_1H-119Sn_ = 25 Hz, 1H, 4-C) 5.39 (d, J_H-H_ = 7 Hz, 1H, 3-C), 2.75 (m, 1H, H-5_a_), 2.23 (m, 1H, H-5_b_), 2.23 (m, 1H, H-6_a_), 1.83 (br, 6H,H-7), 1.78 (s, H-7_b_) 1.62 (s, 9H,H-9), 1.60 (m, 1H,H-6_b_).

**^13^C{^1^H}-NMR** (75.5 MHz, C_6_D_6_, 25 °C): 180 (C-2,^2^*J*_C-F_ = 32 Hz),165.0 (C-4), 134.6 (q, C-1,^1^*J*_C-F_ = 284 Hz), 91.7 (C-3), 58.1 (C-6), 56.1 (C-5), 44.8 (C-7), 44.1(C-7′), 42.7 (C-8), 38.1 (C-9, ^3^*J*_13C-119Sn_ = 24 Hz).

**^19^F-NMR** (282.4 MHz, C_6_D_6_, 25 °C) δ = −74.56 (s, ^1^*J*_F-C_ = 284 Hz, ^2^*J*_F-C_ = 32 Hz).

**^119^Sn{^1^H}-NMR** (111.9 MHz, C_6_D_6_, 25 °C) δ = -157 (s, Δ_1/2_ = 400 Hz)

**Mass spectrometry**

(EI, 20 eV) *m*/*z* (rel. Int., fragment-ion): *L-Sn-SC_4_H_9_*: 418 (10, [M]^+^); 329 (80, [M−SC_4_H_9_]^+^); 209 (2, [M−SnSC_4_H_9_ or -L]^+^).

*Sn(SC_4_H_9_)_4_*: 476 (15, [M]^+^); 387 (24, [M-SC_4_H_9_]^+^); 275 (24, [M−2C_4_H_8_, -SC_4_H_9_]); 241 (8, [M−C_4_H_8_ -2SC_4_H_9_]; 219 (8, [M−SC_4_H_9_ -3C_4_H_8_]); 178 [(SC_4_H_9_)_2_]^+^.

## 4. Conclusions

We report here a new heteroleptic precursor [Sn(SBu*^t^*)(*tfb-dmeda*)] **1**, which was characterized thorougly in the solution- and solid-phase by multi-nuclei NMR and single crystal X-ray structure and studied for thermal and fragmentation behaviors by thermogravimetric and mass spectrometry, respectively. As compared to corresponding homoleptic tin(IV) alkylthiolates, which could not be successfully applied in the CVD process, the heteroleptic configuration of **1** and the resulting increase in the Sn-S bond strength ensures a predictable decomposition pattern for it. So while the tertiarybutyl thiolate ligand decomposes in a neat and clean manner due to the presence of an inherent decomposition mechanism [66], the monoanionic tridentate chelate ligand departs in a clean manner without changing the electronic structure of the Sn(II) center or contaminating the growing SnS film, for example, through probable incorporation of heteroatoms (N, O). This, along with the low precursor temperature, enables deposition of high-quality SnS thin films by CVD under mild conditions. While the use of FTO as substrate generated a preferred orientation of the deposited SnS films along the (040) plane, the films deposited on Mo-coated soda-lime substrate showed no such preference. In both cases, the deposited films were well-crystalline, dense, and of uniform thickness. This work showing the “launch vehicle” role of the auxiliary ligand underlines the potential of design and subtle choice of ligands and coligands in synthesizing efficient molecular precursors for chemical vapor deposition.

## Data Availability

All data presented is available in this manuscript.

## Supplementary Materials

The following are available online: Figure S1: Comparison of the ^1^H-NMR spectra of Htfb-dmeda and **1a** in C_6_D_6_ and recorded at 300 MHz, Figure S2: ^1^H-NMR variable temperature spectra of **1** in toluene-d_8_. The bottom spectrum at T = 298 K was recorded after cooling when the NMR tube was warmed up to room temperature again, showing the full reversibility of the observed equilibria, Figure S3: Enlarged part of the ^1^H-NMR variable temperature spectra of **1** (Figure S2) for better visualization, Figure S4: Variable temperature ^119^Sn-NMR spectra of **1**. The bottom spectrum at T = 298 K was recorded at room temperature, after cooling, showing the initial chemical shift is restituted, Figure S5: ^119^Sn,^1^H-HMBC spectrum of **1**, recorded at 300 MHz, Figure S6: Experimental EI-MS fragmentation pattern of **1**, Figure S7: Experimental EI-MS fragmentation pattern of Sn(SBu^t^)_2_, Table S1: Selected bond lengths of **1**, Table S2: Selected bond angles of **1**.

## Sample Availability

Samples of the compound **1** and SnS thin films obtained by CVD are available from the authors.

## References

[1] Ling X., Wang H., Huang S., Xia F., Dresselhaus M.S. (2015). The Renaissance of Black Phosphorus. Proc. Natl. Acad. Sci. USA.

[2] Huang X., Woo H., Wu P., Hong H.J., Jung W.G., Kim B.-J., Vanel J.-C., Choi J.W. (2017). Simple Eco-Friendly Synthesis of the Surfactant Free SnS Nanocrystal toward the Photoelectrochemical Cell Application. Sci. Rep..

[3] Reddy N.K., Devika M., Prashantha M., Ramesh K., Gunasekhar K.R. (2012). In Situ Structural Studies on Orthorhombic SnS Micro-Crystals. Eur. Phys. J. Appl. Phys..

[4] Ning J., Men K., Xiao G., Wang L., Dai Q., Zou B., Liu B., Zou G. (2010). Facile Synthesis of IV–VI SnS Nanocrystals with Shape and Size Control: Nanoparticles, Nanoflowers and Amorphous Nanosheets. Nanoscale.

[5] Xia J., Li X.-Z., Huang X., Mao N., Zhu D.-D., Wang L., Xu H., Meng X.-M. (2016). Physical Vapor Deposition Synthesis of Two-Dimensional Orthorhombic SnS Flakes with Strong Angle/Temperature-Dependent Raman Responses. Nanoscale.

[6] Wiedemeier H., Georg H., Schnering G. (1978). von Refinement of the Structures of GeS, GeSe, SnS and SnSe. Z. für Krist.-Cryst. Mater..

[7] Manzeli S., Ovchinnikov D., Pasquier D., Yazyev O.V., Kis A. (2017). 2D Transition Metal Dichalcogenides. Nat. Rev. Mater..

[8] Brune V., Grosch M., Weißing R., Hartl F., Frank M., Mishra S., Mathur S. (2021). Influence of the Choice of Precursors on the Synthesis of Two-Dimensional Transition Metal Dichalcogenides. Dalt. Trans..

[9] Shan Y., Li Y., Pang H. (2020). Applications of Tin Sulfide-Based Materials in Lithium-Ion Batteries and Sodium-Ion Batteries. Adv. Funct. Mater..

[10] Wang S.F., Wang W., Fong W.K., Yu Y., Surya C. (2017). Tin Compensation for the SnS Based Optoelectronic Devices. Sci. Rep..

[11] Sarkar A.S., Stratakis E. (2020). Recent Advances in 2D Metal Monochalcogenides. Adv. Sci..

[12] Choi H., Lee N., Park H., Choi Y., Kim K., Choi Y., Kim J., Song S., Yuk H., Jeon H. (2019). Development of a SnS Film Process for Energy Device Applications. Appl. Sci..

[13] Ichimura M., Takeuchi K., Ono Y., Arai E. (2000). Electrochemical Deposition of SnS Thin Films. Thin Solid Films.

[14] Avellaneda D., Nair M.T.S., Nair P.K. (2008). Polymorphic Tin Sulfide Thin Films of Zinc Blende and Orthorhombic Structures by Chemical Deposition. J. Electrochem. Soc..

[15] Mariano A.N., Chopra K.L. (1967). Polymorphism in Some IV-VI Compounds Induced by High Pressure and Thin-Film Epitaxial Growth. Appl. Phys. Lett..

[16] Gao C., Shen H., Sun L. (2011). Preparation and Properties of Zinc Blende and Orthorhombic SnS Films by Chemical Bath Deposition. Appl. Surf. Sci..

[17] Polivtseva S., Katerski A., Kärber E., Oja Acik I., Mere A., Mikli V., Krunks M. (2017). Post-Deposition Thermal Treatment of Sprayed SnS Films. Thin Solid Films.

[18] Jain P., Arun P. (2013). Influence of Grain Size on the Band-Gap of Annealed SnS Thin Films. Thin Solid Films.

[19] Zhang H., Balaji Y., Mehta A.N., Heyns M., Caymax M., Radu I., Vandervorst W., Delabie A. (2018). Formation Mechanism of 2D SnS_2_ and SnS by Chemical Vapor Deposition Using SnCl_4_ and H_2_S. J. Mater. Chem. C.

[20] Price L.S., Parkin I.P., Hardy A.M.E., Clark R.J.H., Hibbert T.G., Molloy K.C. (1999). Atmospheric Pressure Chemical Vapor Deposition of Tin Sulfides (SnS, Sn_2_S_3_, and SnS_2_) on Glass. Chem. Mater..

[21] Barone G., Hibbert T.G., Mahon M.F., Molloy K.C., Price L.S., Parkin I.P., Hardy A.M.E.E., Field M.N. (2001). Deposition of Tin Sulfide Thin Films from Tin(Iv) Thiolate Precursors. J. Mater. Chem..

[22] Park J.H., Kang S.G., Lee Y.K., Chung T.M., Park B.K., Kim C.G. (2020). Tin(II) Aminothiolate and Tin(IV) Aminothiolate Selenide Compounds as Single-Source Precursors for Tin Chalcogenide Materials. Inorg. Chem..

[23] Park J., Song M., Jung W.M., Lee W.Y., Lee J., Kim H., Shim I.W. (2012). Preparation of SnS Thin Films by MOCVD Method Using Single Source Precursor, Bis(3-Mercapto-1-Propanethiolato) Sn(II). Bull. Korean Chem. Soc..

[24] Robinson F., Curran P.J., de Groot C.H., Hardie D., Hector A.L., Holloway K., Huang R., Newbrook D., Reid G. (2021). ^n^Bu_2_Sn(SnBu)_2_ and ^n^Bu_3_SnE^n^Bu (E = S or Se) – Effective Single Source Precursors for the CVD of SnS and SnSe Thermoelectric Thin Films. Mater. Adv..

[25] Catherall A.L., Harris S., Hill M.S., Johnson A.L., Mahon M.F. (2017). Deposition of SnS Thin Films from Sn(II) Thioamidate Precursors. Cryst. Growth Des..

[26] Kevin P., Lewis D.J., Raftery J., Azad Malik M., O’Brien P. (2015). Thin Films of Tin(II) Sulphide (SnS) by Aerosol-Assisted Chemical Vapour Deposition (AACVD) Using Tin(II) Dithiocarbamates as Single-Source Precursors. J. Cryst. Growth.

[27] Ramasamy K., Kuznetsov V.L., Gopal K., Malik M.A., Raftery J., Edwards P.P., O’Brien P. (2013). Organotin Dithiocarbamates: Single-Source Precursors for Tin Sulfide Thin Films by Aerosol-Assisted Chemical Vapor Deposition (AACVD). Chem. Mater..

[28] Bade B.P., Garje S.S., Niwate Y.S., Afzaal M., O’Brien P. (2008). Tribenzyltin(IV)Chloride Thiosemicarbazones: Novel Single Source Precursors for Growth of SnS Thin Films. Chem. Vap. Depos..

[29] Ahmet I.Y., Guc M., Sánchez Y., Neuschitzer M., Izquierdo-Roca V., Saucedo E., Johnson A.L. (2019). Evaluation of AA-CVD Deposited Phase Pure Polymorphs of SnS for Thin Films Solar Cells. RSC Adv..

[30] Ahmet I.Y., Hill M.S., Johnson A.L., Peter L.M. (2015). Polymorph-Selective Deposition of High Purity SnS Thin Films from a Single Source Precursor. Chem. Mater..

[31] Kana A.T., Hibbert T.G., Mahon M.F., Molloy K.C., Parkin I.P., Price L.S. (2001). Organotin Unsymmetric Dithiocarbamates: Synthesis, Formation and Characterisation of Tin(II) Sulfide Films by Atmospheric Pressure Chemical Vapour Deposition. Polyhedron.

[32] Hibbert T.G., Mahon M.F., Molloy K.C., Price L.S., Parkin I.P. (2001). Deposition of Tin Sulfide Thin Films from Novel, Volatile (Fluoroalkythiolato)Tin(IV) Precursors. J. Mater. Chem..

[33] Parkin I.P., Price L.S., Hibbert T.G., Molloy K.C. (2001). The First Single Source Deposition of Tin Sulfide Coatings on Glass: Aerosol-Assisted Chemical Vapour Deposition Using [Sn(SCH_2_CH_2_S)_2_]. J. Mater. Chem..

[34] Frank M., Jürgensen L., Leduc J., Stadler D., Graf D., Gessner I., Zajusch F., Fischer T., Rose M.-A., Mueller D.N. (2019). Volatile Rhenium(I) Compounds with Re–N Bonds and Their Conversion into Oriented Rhenium Nitride Films by Magnetic Field-Assisted Vapor Phase Deposition. Inorg. Chem..

[35] Harris D.H., Lappert M.F. (1974). Monomeric, Volatile Bivalent Amides of Group IVB Elements, M(NR^1^_2_)_2_ and M(NR_1_R_2_)_2_ (M = Ge, Sn, or Pb; R_1_ = Me_3_Si, R_2_ = Me_3_C). J. Chem. Soc. Chem. Commun..

[36] Mantina M., Chamberlin A.C., Valero R., Cramer C.J., Truhlar D.G. (2009). Consistent van Der Waals Radii for the Whole Main Group. J. Phys. Chem. A.

[37] Pyykkö P., Atsumi M. (2009). Molecular Single-Bond Covalent Radii for Elements 1–118. Chem. Eur. J..

[38] Veith M., Hobein P., Rösler R. (1989). Cyclische Diazastannylene, XXX [1] Symmetrisch Und Asymmetrisch Substituierte German- Und Stannandiyle Mit Amid-, Alkoholat- Und Thiolat-Liganden, Teil I [2]. Z. fur Naturforsch.-Sect. B J. Chem. Sci..

[39] Hitchcock P.B., Lappert M.F., Samways B.J., Weinberg E.L. (1983). Metal (Li, GeII, GeIII, SnII, and PbII) 2,6-Dialkylbenzenethiolates; X-Ray Crystal Structures of Sn(SAr)_2_ (Ar = C_6_H_2_Bu^t^_3_-2,4,6) and [M(SAr)_2_]_3_ (M = Sn or Pb, Ar = C_6_H_3_Pr^i^_2_-2,6). J. Chem. Soc. - Chem. Commun..

[40] Khrustalev V.N., Portnyagin I.A., Zemlyansky N.N., Borisova I.V., Nechaev M.S., Ustynyuk Y.A., Antipin M.Y., Lunin V. (2005). New Stable Germylenes, Stannylenes, and Related Compounds: 6. Heteroleptic Germanium(II) and Tin(II) Compounds [(SiMe_3_)_2_N-E^14^-OCH_2_CH_2_NMe_2_]_n_ (E^14^ = Ge, n = 1; Sn, n = 2): Synthesis and Structure. J. Organomet. Chem..

[41] Zemlyanskii N.N., Borisova I.V., Kuznetsova M.G., Khrustalev E.N., Antipin M.Y., Ustynyuk Y.A., Lunin E.E., Eaborn C., Hill M.S., Smith J.D. (2003). New Stable Germylenes, Stannylenes, and Related Compounds II. Bis(Butylthio)Tin(II) and Ate-Complexes [(Me_3_Si)_3_CE(μ-SBu)_2_Li(THF)_2_] (E = Ge, Sn). Synthesis and Structure. Russ. J. Org. Chem..

[42] Eichhöfer A., Jiang J.J., Sommer H., Weigend F., Fuhr O., Fenske D., Su C.Y., Buth G. (2010). 1-D-Tin(II) Phenylchalcogenolato Complexes _∞1_[Sn(EPh)_2_] (E = S, Se, Te) - Synthesis, Structures, Quantum Chemical Studies and Thermal Behaviour. Eur. J. Inorg. Chem..

[43] Rekken B.D., Brown T.M., Fettinger J.C., Lips F., Tuononen H.M., Herber R.H., Power P.P. (2013). Dispersion Forces and Counterintuitive Steric Effects in Main Group Molecules: Heavier Group 14 (Si–Pb) Dichalcogenolate Carbene Analogues with Sub-90° Interligand Bond Angles. J. Am. Chem. Soc..

[44] Dean P.A.W., Vittal J.J., Payne N.C. (1985). Syntheses and X-Ray Structural Analyses of [(C_6_H_5_)_4_As][Sn(EC_6_H_5_)_3_], E = S and Se. Can. J. Chem..

[45] Barone G., Hibbert T.G., Mahon M.F., Molloy K.C., Parkin I.P., Price L.S., Silaghi-Dumitrescu I. (2001). Structural Distortions in Homoleptic (RE)_4_A (E = O, S, Se; A = C, Si, Ge, Sn): Implications for the CVD of Tin Sulfides. J. Chem. Soc. Dalt. Trans..

[46] Olmstead M.M., Power P.P. (1984). Structural Studies of Tin(II) and Lead(II) Dimethylamides: X-Ray Crystal Structure of [Sn(NMe_2_)_2_]_2_ and Isolation of Its Lead Analogue. Inorg. Chem..

[47] Zemlyansky N.N., Borisova I.V., Kuznetsova M.G., Khrustalev V.N., Ustynyuk Y.A., Nechaev M.S., Lunin V.V., Barrau J., Rima G. (2003). New Stable Germylenes, Stannylenes, and Related Compounds. 1. Stable Germanium(II) and Tin(II) Compounds M(OCH_2_CH_2_NMe_2_)_2_ (M = Ge, Sn) with Intramolecular Coordination Metal-Nitrogen Bonds. Synthesis and Structure. Organometallics.

[48] Holloway C.E., Melnik M. (1998). Tin Coordination Compounds: Classification and Analysis of Crystallographic and Structural Data. Main Gr. Met. Chem..

[49] Persson I., D’Angelo P., Lundberg D. (2016). Hydrated and Solvated Tin(II) Ions in Solution and the Solid State, and a Coordination Chemistry Overview of the D10s2 Metal Ions. Chem. Eur. J..

[50] Wang L., Kefalidis C.E., Roisnel T., Sinbandhit S., Maron L., Carpentier J.-F., Sarazin Y. (2014). Structure vs ^119^Sn NMR Chemical Shift in Three-Coordinated Tin(II) Complexes: Experimental Data and Predictive DFT Computations. Organometallics.

[51] Du Mont W.-W., Grenz M. (1985). Dimere Phospha- Und Thiastannylene: Ylidartige Diphospha- Und Dithiadistannetane. Chem. Ber..

[52] Pieper N., Klaus-Mrestani C., Schürmann M., Jurkschat K., Biesemans M., Verbruggen I., Martins J.C., Willem R. (1997). Synthesis, Molecular Structure, and Stereochemical Nonrigidity of Bis(3-(Dimethylamino)Propyl)Difluorostannane Dihydrate, {[Me_2_N(CH_2_)_3_]_2_ SnF_2_ ·2H_2_O}, and Enhanced Reactivity of Its Fluoride Adduct {[Me_2_N(CH_2_)_3_]_2_SnF_3_}-Bu_4_N^+^ Toward Dichloromethane. Organometallics.

[53] Meurice J.C., Duboudin J.G., Ratier M., Pétraud M., Willem R., Biesemans M. (1999). Conformational Investigations of Ester-Functionalized Gem-Distannyl Derivatives by ^1^H-^119^Sn Correlation NMR. Organometallics.

[54] Martins J.C., Biesemans M., Willem R. (2000). Tin NMR Based Methodologies and Their Use in Structural Tin Chemistry. Prog. Nucl. Magn. Reson. Spectrosc..

[55] Janzen A.F., Vaidya O.C., Willis C.J. (1981). Reaction of Arsenic, Antimony, Bismuth and Sulfur Fluorides with (t-Butylthio) Trimethylsilane. J. Inorg. Nucl. Chem..

[56] Steinmann V., Chakraborty R., Rekemeyer P.H., Hartman K., Brandt R.E., Polizzotti A., Yang C., Moriarty T., Gradečak S., Gordon R.G. (2016). A Two-Step Absorber Deposition Approach To Overcome Shunt Losses in Thin-Film Solar Cells: Using Tin Sulfide as a Proof-of-Concept Material System. ACS Appl. Mater. Interfaces.

[57] Revathi N., Bereznev S., Iljina J., Safonova M., Mellikov E., Volobujeva O. (2013). PVD Grown SnS Thin Films onto Different Substrate Surfaces. J. Mater. Sci. Mater. Electron..

[58] Sutter P., Sutter E. (2018). Growth Mechanisms of Anisotropic Layered Group IV Chalcogenides on van Der Waals Substrates for Energy Conversion Applications. ACS Appl. Nano Mater..

[59] Lee H., Yang W., Tan J., Park J., Shim S.G., Park Y.S., Yun J.W., Kim K.M., Moon J. (2020). High-Performance Phase-Pure SnS Photocathodes for Photoelectrochemical Water Splitting Obtained via Molecular Ink-Derived Seed-Assisted Growth of Nanoplates. ACS Appl. Mater. Interfaces.

[60] Sheldrick G.M. (2008). A Short History of SHELX. Acta Crystallogr. Sect. A Found. Crystallogr..

[61] Sheldrick G.M. (2015). SHELXT—Integrated Space-Group and Crystal-Structure Determination. Acta Crystallogr. Sect. A Found. Crystallogr..

[62] Hübschle C.B., Sheldrick G.M., Dittrich B. (2011). ShelXle: A Qt Graphical User Interface for SHELXL. J. Appl. Crystallogr..

[63] Schläfer J., Graf D., Fornalczyk G., Mettenbörger A., Mathur S. (2016). Fluorinated Cerium(IV) Enaminolates: Alternative Precursors for Chemical Vapor Deposition of CeO2 Thin Films. Inorg. Chem..

[64] Müller R., Hernandez-Ramirez F., Shen H., Du H., Mader W., Mathur S. (2012). Influence of Precursor Chemistry on Morphology and Composition of CVD-Grown SnO_2_ Nanowires. Chem. Mater..

[65] Graf D., Schläfer J., Garbe S., Klein A., Mathur S. (2017). Interdependence of Structure, Morphology, and Phase Transitions in CVD Grown VO_2_ and V_2_O_3_ Nanostructures. Chem. Mater..

[66] Gahlot S., Purohit B., Jeanneau E., Mishra S. (2021). Coinage Metal Complexes with Di-Tertiary-Butyl Sulfide as Precursors with Ultra-Low Decomposition Temperature. Chem. Eur. J..

