# Supplementary material for: Vapor Phase Synthesis of SnS Facilitated by Ligand-Driven “Launch Vehicle” Effect in Tin Precursors
Source: Molecules. 2021 Sep 3;26(17):5367. doi: 10.3390/molecules26175367 (PMC8433875; doi:10.3390/molecules26175367)
Supplement: Supplementary file 1 [file molecules-26-05367-s001.zip › molecules-1356699-supplementary.pdf]

Supporting Information

Vapor phase synthesis of SnS facilitated by ligand-driven 'launch vehicle' effect in tin precursors

Ufuk Atamtürk[1], Veronika Brune[1], Shashank Mishra[2,*] and Sanjay Mathur[1,*]

[1]Institute of Inorganic Chemistry, Greinstraße 6, 50939 Cologne, Germany
[2]Université Claude Bernard Lyon 1, CNRS, UMR 5256, IRCELYON, 2 avenue Albert Einstein, 69626 Villeurbanne, France
Correspondence:
shashank.mishra@ircelyon.univ-lyon1.fr (S. Mishra)
sanjay.mathur@uni-koeln.de (S. Mathur)

NMR Spectroscopy

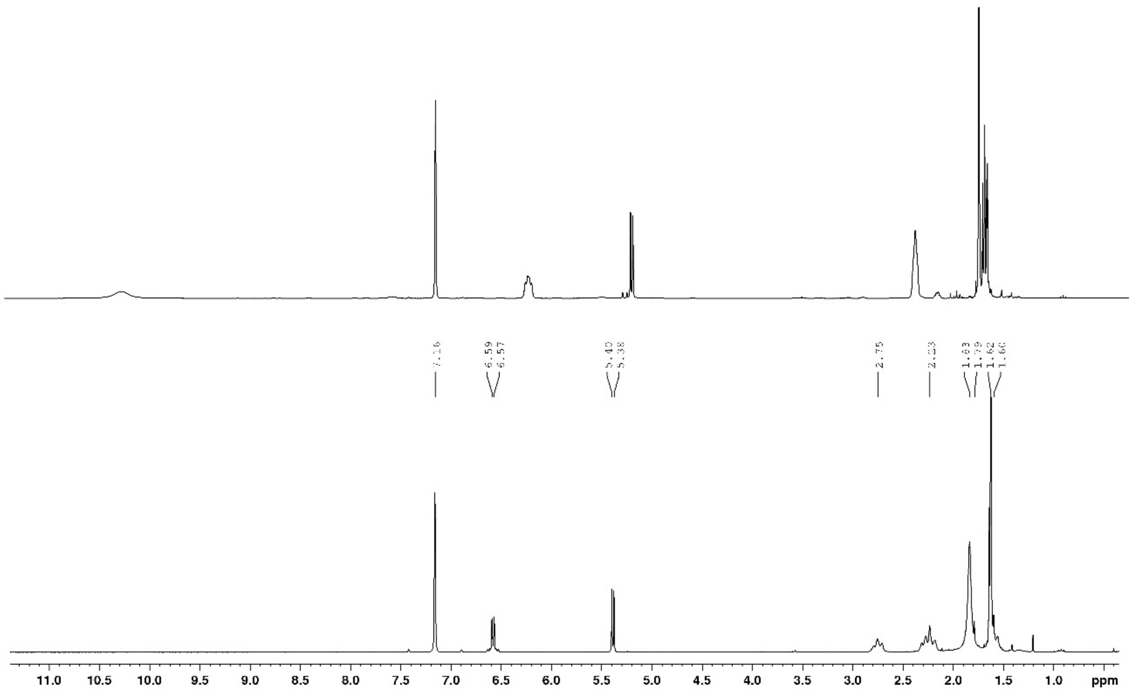

*Fig. S1: Comparison of the ^{1}H NMR spectra of Htfb-dmeda and **1**a in C_6D_6 and recorded at 300 MHz.*

Variable temperature NMR

Variable temperature NMR spectra were recorded on a AVANCE 400 spectrometer equipped with a 5 mm TBI probehead. The cooling rate was adjusted so that lowering the temperature by 10 K was reached in 20 min of time. At each temperature the sample was allowed to equilibrate for 10-15 min, followed by recalibrating the frequencies the by the tune and match procedure and shimming of the magnets. The ^{119}Sn{^{1}H}-NMR spectra were offset to o1p = -157, and the spectral widths were narrowed to sw = 500. The minimum number of scans for ^{119}Sn{^{1}H} was ns = 128 and the max. ns = 1 k (depending on the temperature stability the VT unit of the spectrometers was able to maintain) and in all ^{1}H-NMR spectra ns = 32.

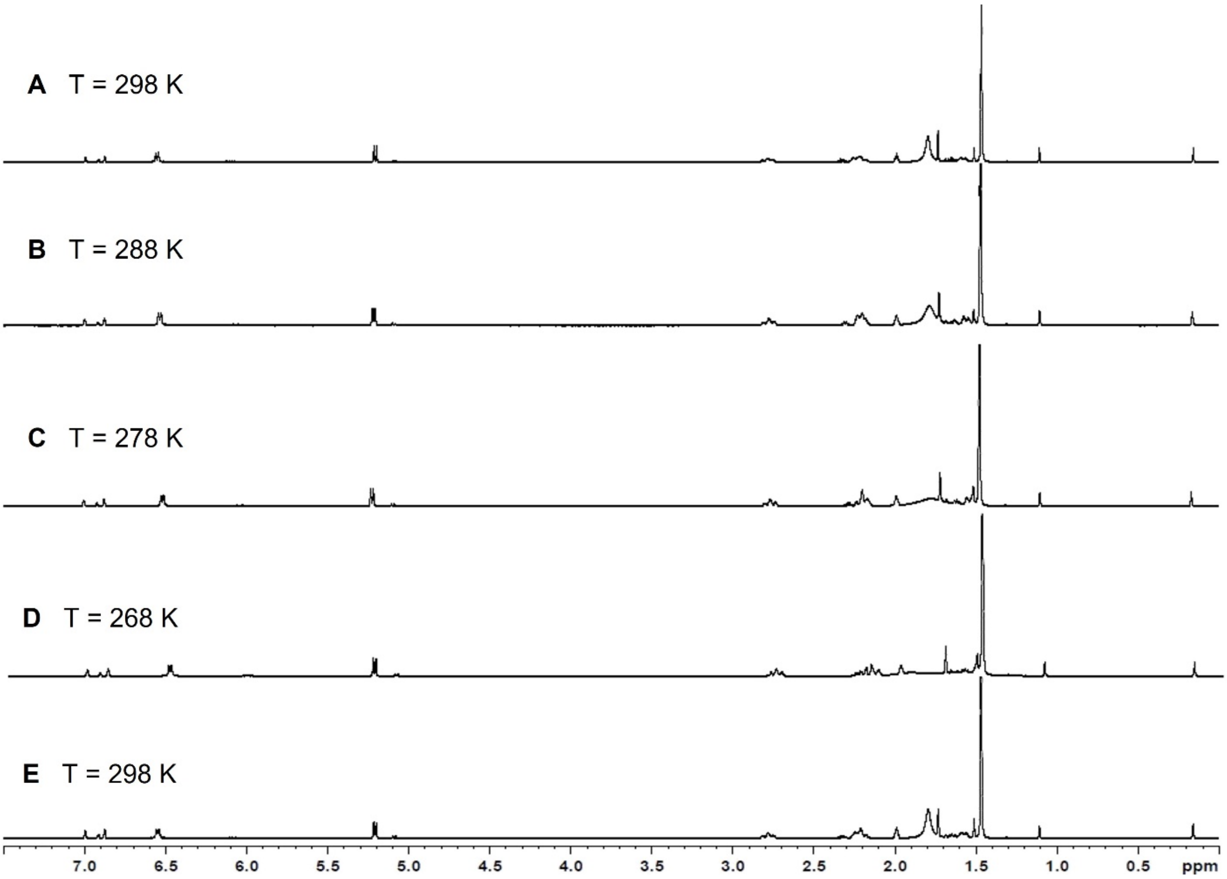

Fig. S2: ^{1}H-NMR variable temperature spectra of **1** *in toluene-d$_8$. The bottom spectrum at T = 298 K was recorded after cooling, when the NMR tube was warmed up to room temperature again, showing the full reversibility of the observed equilibria.*

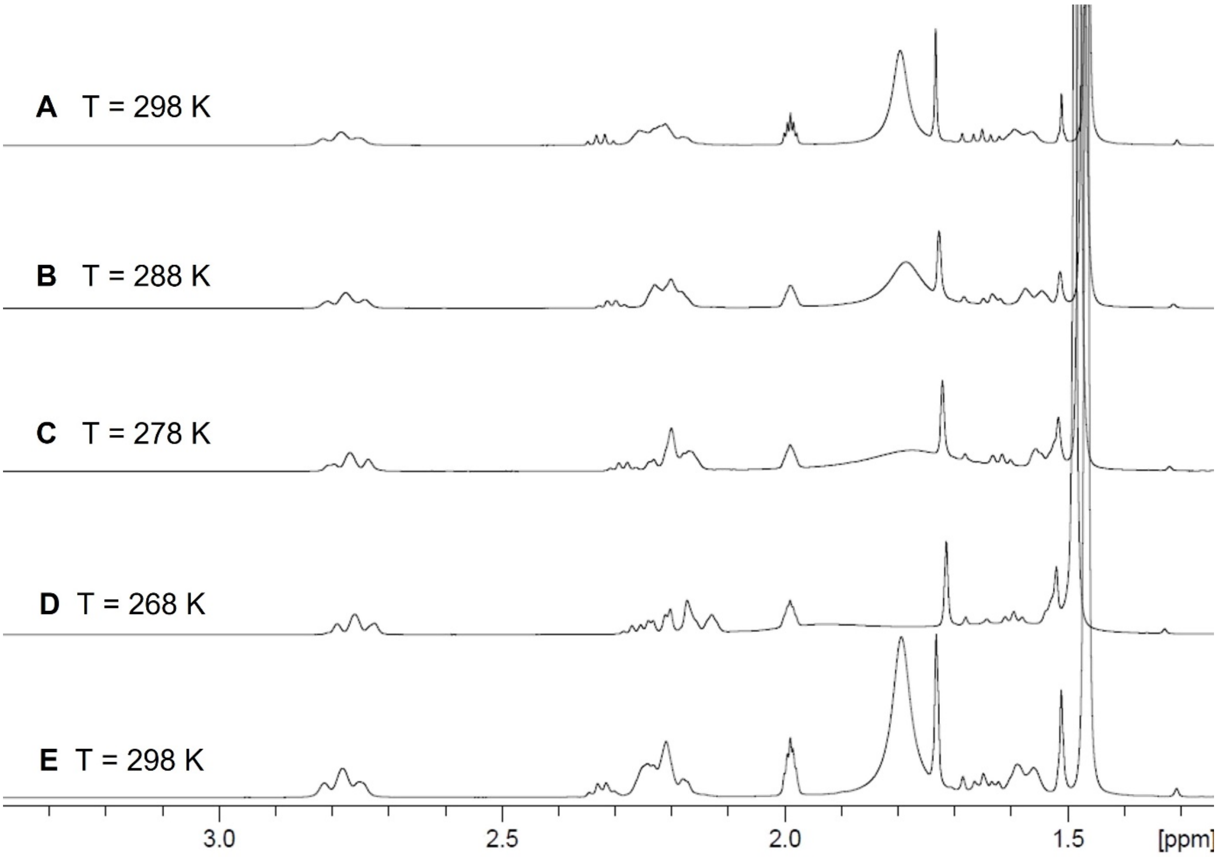

*Fig. S3: Enlarged part of the ¹H-NMR variable temperature spectra of **1** (Fig. S2) for better visualization.*

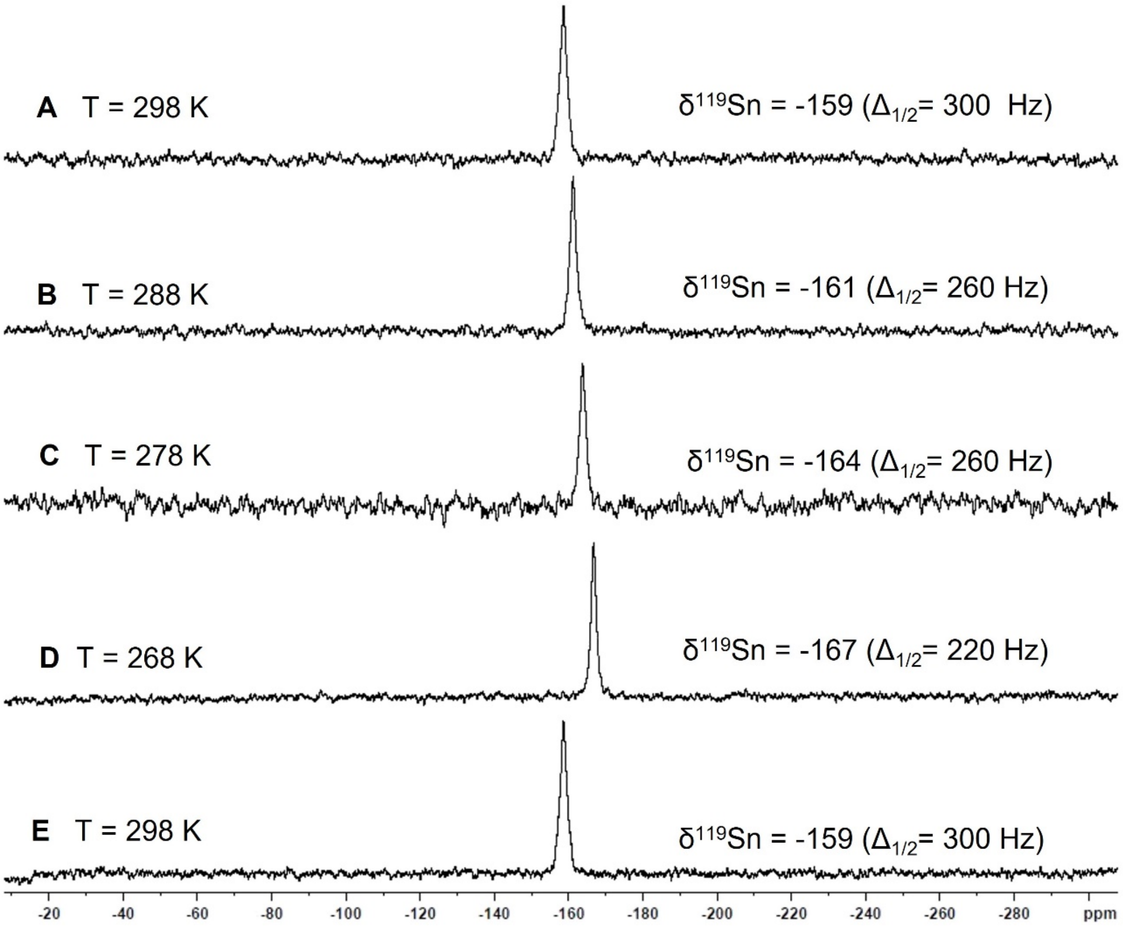

*Fig. S4: Variable temperature ^{119}Sn-NMR spectra of **1.** The bottom spectrum at T = 298 K was recorded at room temperature, after cooling, showing the initial chemical shift is restituted.*

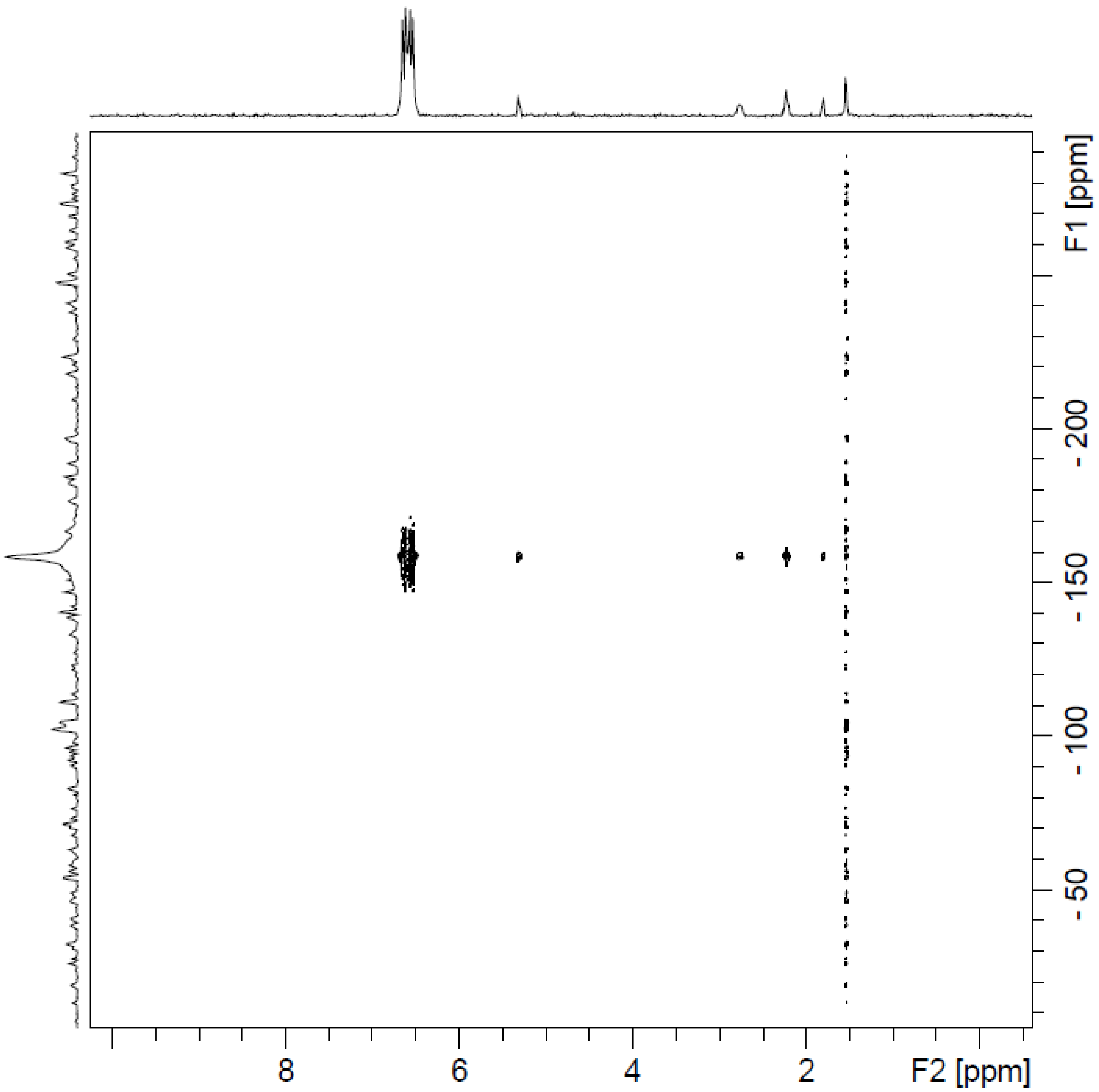

*Fig. S5: ^{119}Sn,^{1}H-HMBC spectrum of **1**, recorded at 300 MHz.*

Mass spectrometry

For Sn(SBu^t)$_4$ the literature reported[1] chemical formulae of the fragment ions is listed below. No rel. intensity and corresponding m/z values were given, the latter was added manually for comparison.

Sn(SBu^t)$_4$: [Sn(SC$_4$H$_9$)$_4$]$^+$ ([M]$^+$, *m/z* 476), [Sn(S)(SC$_4$H$_9$)$_3$]$^+$ (*m/z* 419), [Sn(SC$_4$H$_9$)$_3$]$^+$ (*m/z* 387), [Sn(SH)(SC$_4$H$_9$)$_2$]$^+$ (*m/z* 331), [Sn(SH)$_2$(SC$_4$H$_9$)]$^+$ (*m/z* 275), [Sn(SH)$_3$]$^+$ (*m/z* 219)

The fragment ion series (fig.1a,b) generated by loss of -C$_4$H$_9$ (56 u) is accounting for a large part of the mass spectra observed for **1** : [Sn(SC$_4$H$_9$)$_4$]$^+$ (*m/z* 476), [Sn(SC$_4$H$_9$)$_3$]$^+$ (*m/z* 387), [Sn(SH)(SC$_4$H$_9$)$_2$]$^+$ (*m/z* 331), [(Sn(SH)$_2$(SC$_4$H$_9$)]$^+$ (*m/z* 275), [(Sn(S)(SC$_4$H$_9$)]$^+$ (*m/z* 241), [Sn(SH)$_3$]$^+$ (*m/z* 219).)

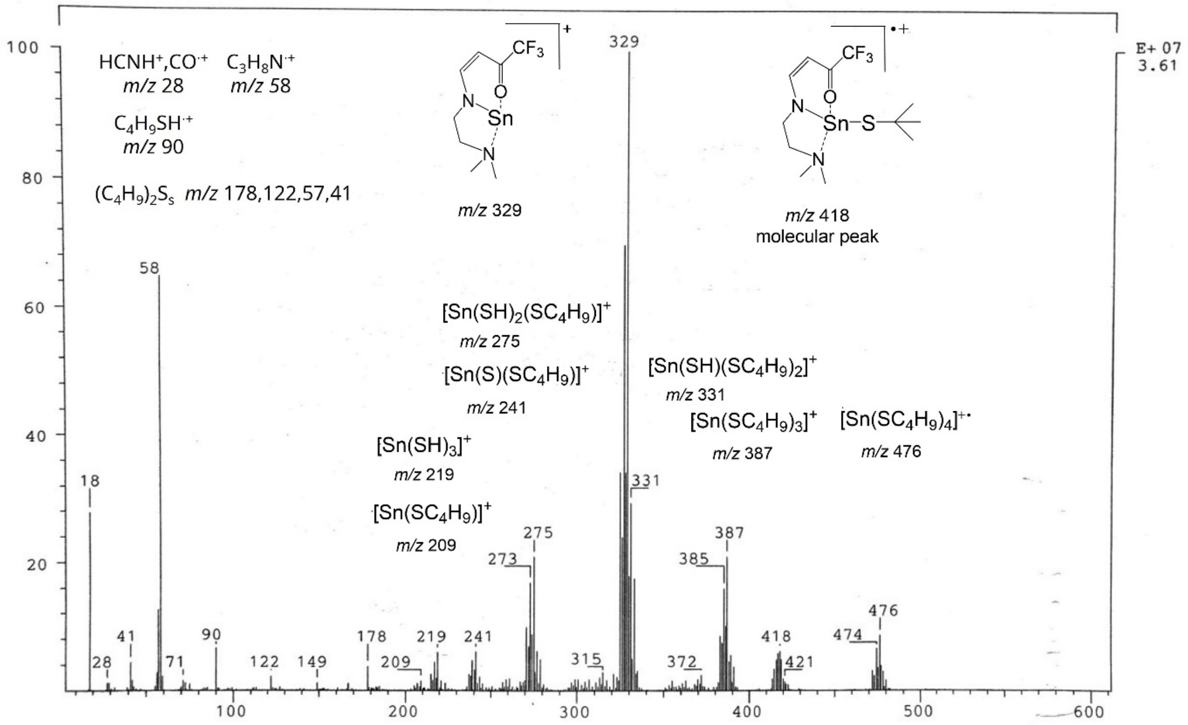

Fig. S6: Experimental EI-MS fragmentation pattern of ***1.***

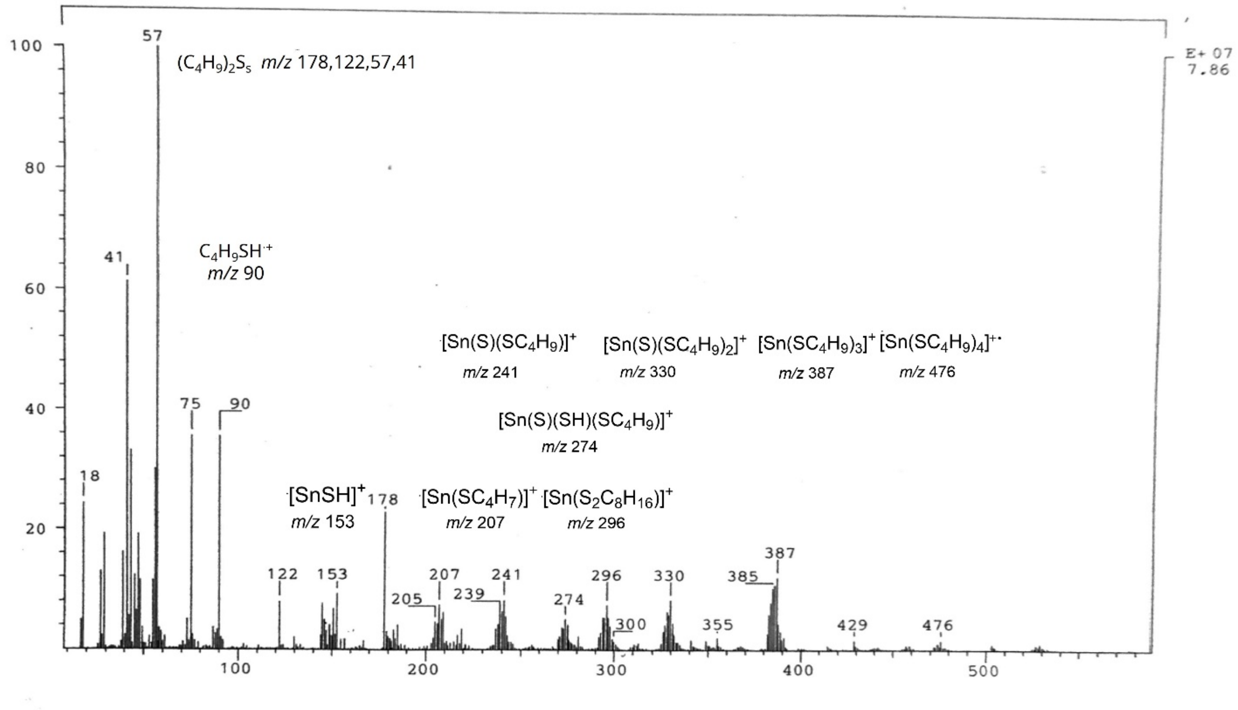

Fig. S7: Experimental EI-MS fragmentation pattern of Sn(SBu^t)$_2$.

For Sn(SBu^t)$_2$ the molecular peak calculated as [Sn(SC$_4$H$_9$)$_2$]$^+$ (*m/z* 298) is absent. Two fragment ions with the corresponding chemical formulae [(Sn(SC$_4$H$_7$)]$^+$ (*m/z* 207) and [(Sn(SC$_8$H$_{16}$)]$^+$ (*m/z* 296) point towards the decomposition of a dimer, calculated

as $[(Sn(SC_4H_9)_2)_2]^+$ (*m/z* 596), which requires the analysis at an extended detection range to be able to draw further conclusions.

Table S1: Selected bond lengths of ***1***

	Bond lengths / pm		Bond lengths / pm
Sn(1)-S(11)	248.61(8)	Sn(1)-O(11)	226.9(2)
Sn(2)-S(21)	249.25(8)	Sn(2)-O(21)	224.8(2)
Sn(3)-S(31)	248.89(8)	Sn(3)-O(31)	223.7(2)
Sn(4)-S(41)	249.27(8)	Sn(4)-O(41)	225.4(2)
Sn(1)-N(11)	224.9(2)	Sn(3)-N(31)	226.0(2)
Sn(1)-N(12)	254.2(3)	Sn(3)-N(32)	254.1(3)
Sn(2)-N(21)	224.6(3)	Sn(4)-N(41)	224.2(2)
Sn(2)-N(22)	254.5(3)	Sn(4)-N(42)	255.7(3)

Table S2: Selected bond angles of ***1***

	Bond angles / °		Bond angles / °
N(11)-Sn(1)-S(11)	84.93(7)	O(11)-Sn(1)-S(11)	90.77(6)
N(21)-Sn(2)-S(21)	83.85(7)	O(21)-Sn(2)-S(21)	91.46(6)
N(31)-Sn(3)-S(31)	83.12(6)	O(31)-Sn(3)-S(31)	92.53(6)
N(41)-Sn(4)-S(41)	84.33(7)	O(41)-Sn(4)-S(41)	91.01(6)
N(12)-Sn(1)-S(11)	88.26(7)	O(11)-Sn(1)-N(12)	154.04(8)
N(22)-Sn(2)-S(21)	86.47(7)	O(21)-Sn(2)-N(22)	154.10(9)
N(32)-Sn(3)-S(31)	87.10(6)	O(31)-Sn(3)-N(32)	153.01(8)
N(42)-Sn(4)-S(41)	87.96(6)	O(41)-Sn(4)-N(42)	153.76(9)

[1] A. F. Janzen, O. C. Vaidya, C. J. Willis, *J. Inorg. Nucl. Chem.* **1981**, *43*, 1469–1471.
